# Key bacterial taxa determine longitudinal dynamics of aromatic amino acid catabolism in infants’ gut

**DOI:** 10.1080/19490976.2023.2221426

**Published:** 2023-06-25

**Authors:** Martin F. Laursen, Anurag K. Sinha, Mikael Pedersen, Henrik M. Roager

**Affiliations:** a National Food Institute, Technical University of Denmark, Lyngby, Denmark; b Department of Nutrition, Exercise and Sports, University of Copenhagen, Frederiksberg, Denmark

**Keywords:** Infant, gut microbiota, metabolites, short chain fatty acids, aromatic amino acid catabolites, longitudinal sampling

## Abstract

The development of the gut microbiota in early life is linked to metabolic, neuronal, and immunological development. Recent studies have shown that bacterial production of short-chain fatty acids (SCFAs) and aromatic amino acid (AAA) catabolites in the gut can mediate host–microbe interactions. However, the dynamics of these microbiota-derived metabolites and the key bacterial taxa producing AAA catabolites during infancy are largely unknown. Here, we investigated the longitudinal dynamics of the microbiota and microbiota-derived SCFAs and AAA catabolites in more than 200 fecal samples from 25 healthy breast- or mixed-fed Danish infants during the first 6 months of life. We found that the gut microbiota composition and metabolism were highly individual but showed significant development over time. SCFAs and specific groups of AAA catabolites showed distinct temporal abundance patterns. Furthermore, we identified bacterial taxa responsible for the generation of AAA catabolites by associating the dynamics of gut microbial taxa and AAA catabolites and subsequently validating these associations in vitro by cultivation of strains representing the associated taxa. In addition to specific *Bifidobacterium* species being the main producers of aromatic lactic acids, we identified *Peptostreptococcus anaerobius* as the main producer of aromatic propionic acids, *Ruminococcus gnavus* as a main producer of tryptamine, and *Enterococcus* species as main tyramine producers in infants’ gut. Thus, our results showcase the temporal dynamics of key gut microbial metabolites in early life and demonstrate that the appearance and abundance of specific AAA catabolites result from the appearance and abundance of specific key bacterial taxa in infants’ gut.

## Introduction

Mounting evidence suggests that the development of the gut microbiota is linked to metabolic, neuronal, and immunological development in early life.^1–3^ Yet, the underlying mechanisms and mediators of these host–microbe interactions remain largely unknown.^4^ One way by which gut microbes contribute to host physiology is through the production of a myriad of metabolites.^5^ Therefore, there is an urgent need to study and understand the early-life dynamics of microbiota-metabolites and identify the key bacterial taxa responsible for the production of metabolites vital for host–microbe interactions^6^. So far, most studies have in the context of early life focused on the microbiota-derived short-chain fatty acids (SCFAs) including acetate, propionate, and butyrate, which are key metabolites affecting several physiological processes.^7^ However, we recently demonstrated that aromatic lactic acids, which are produced from the aromatic amino acids (AAAs) by specific breastmilk-promoted *Bifidobacterium* species encoding an aromatic lactate dehydrogenase, may affect immune function in early life.^8^ This emphasizes that microbiota-derived AAA catabolites could also be important for health in the context of early life. Indeed, recent studies have shown that AAA catabolites including tryptophan-derived indoles^9^ may influence host metabolism,^10–12^ fortify the intestinal barrier,^10,13^ protect against pathogenic infections,^14,15^ and exhibit neuroprotective activity.^16–18^ However, at present, the dynamics of microbiota-derived AAA catabolites and the key bacterial taxa producing these metabolites during early infancy remain largely unexplored. Here, we investigated the dynamics in the gut microbiota and abundance of microbiota-derived SCFAs and AAA catabolites in more than 200 fecal samples from 25 healthy breastfed or mixed-fed infants during the first 6 months of life. Our data show that both SCFAs and specific groups of AAA catabolites show distinct temporal abundance patterns. We further evaluated the associations between the microbiota-derived AAA catabolites and the gut microbiota and identified key bacterial taxa that produce different AAA catabolites in infants’ gut.

## Results

### Cohort characteristics

The Copenhagen Infant Gut (CIG) cohort consists of 25 breastfed or mixed-fed infants, longitudinally fecal sampled (9–11 time points per individual) during the first 6 months of life (Table 1). The cohort had an almost equal gender distribution, with 23 of the 25 infants born vaginally and full-term (a set of monozygotic twins were born pre-term and these were the only infants delivered by cesarean section). Besides two sets of monozygotic twins, all remaining infants were singletons. All infants initiated breastfeeding, and only three infants discontinued breastfeeding and shifted to formula milk before the last sampling at 6 months of age. However, for 12 infants, breastfeeding was supplemented with formula milk to various degrees (Table 1). Fifteen infants had introduced small amounts of solid foods between 4 and 6 months of age. Three infants had consumed oral antibiotics only at a single time point during the sampling period. Due to the limited and overlapping data on the mode and the time of birth, as well as few samples associated with the use of antibiotics, we did not analyze these factors in relation to the gut microbiota and metabolome. Since only three infants discontinued breastfeeding and the consistent supplementation of formula milk was confined to four infants (amounts were not recorded) and since the amount of solid food introduced at this age of sampling was still very limited, our study cohort was, unfortunately, not well suited for assessing the effects of these dietary parameters on the gut microbiome and metabolome. Therefore, we focused our analyses on describing the longitudinal dynamics in the gut microbiota and metabolome in mostly breastfed cohort of infants and identifying the key bacterial taxa producing AAA catabolites in this context.

**Table 1.** Cohort characteristics of the Copenhagen Infant Gut study (*n* = 25).

Parameter	*n* (%)
Gender	
*Male*	12 (48%)
*Female*	13 (52%)
Birth classification	
*Full-term*	23 (92%)
*Pre-term*	2 (8%)
Mode of birth	
*Vaginal*	23 (92%)
*C-section*	2 (8%)
Singleton versus twins	
*Singletons*	21 (84%)
*Monozygotic twins*	4 (16%)
Breastfeeding discontinuation	
*>6 months*	22 (88%)
*<6 months*	3 (12%)
Percent of sampling points with recorded exposure to formula^1^	
*0%*	13 (52%)
*1–10%*	6 (24%)
*10–30%*	2 (8%)
*30–50%*	0 (0%)
*50–70%*	1 (4%)
*70–90%*	0 (0%)
*90–100%*	3 (12%)
Solid foods introduced (4–6 months)	
*Yes*	15 (60%)
*No*	10 (40%)
Oral antibiotics ever	
*Yes*	3 (12%)
*No*	22 (88%)

^1^For most individuals, 11 samples were obtained; however, for four infants only ten samples and for one infant only nine samples were obtained during the first 6 months of life.

### Gut microbiota development during the first 6 months of life

**Figure 1.**
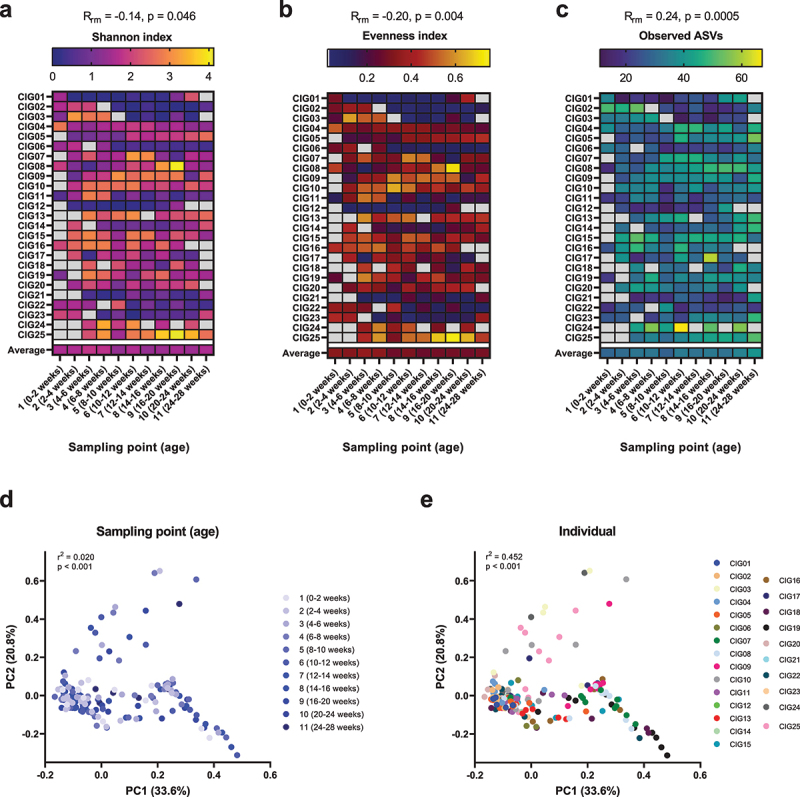
Alpha and beta diversity of the gut microbiota in the CIG cohort. (a–c) Heatmaps showing the alpha diversity measures Shannon index, Evenness index, and Observed ASVs, with gray color indicating missing samples. Statistical significance was evaluated based on repeated-measures correlations, with *R*_rm_ indicating the correlation coefficient between infant age and the indicated alpha diversity measure. (d,e) PCoA plots based on weighted Unifrac distances, colored by (d) sampling point and (e) subject ID. Statistical significance was evaluated based on PERMANOVA. Full color available online.

We assessed the gut microbiota development of the CIG cohort infants by 16S rRNA amplicon sequencing of DNA extracted from the longitudinally sampled feces. In terms of microbial alpha diversity, we observed on average a slight decrease in Shannon index, driven by a significant, but a still modest decrease in evenness over the course of the first six months of life (Figure 1a-b). Conversely, we found a significant, but modest increase in amplicon sequence variant (ASV) richness with age, essentially driven by the last two sampling points (Figure 1c). However, for all alpha diversity measures, strong individual patterns were observed. In terms of beta diversity, as measured by weighted Unifrac, a weak but significant effect of age, explaining 2.0% of community variation, was found (Figure 1d). A much more pronounced effect of the individual was observed, explaining 45.2% of community variation (Figure 1e). These results highlight that longitudinal changes in the infant gut microbiota are highly individual and emphasizes the need to investigate intra-individual connections between the gut microbiota and metabolome.

### Longitudinal dynamics of SCFAs and AAA catabolites

**Figure 2.**
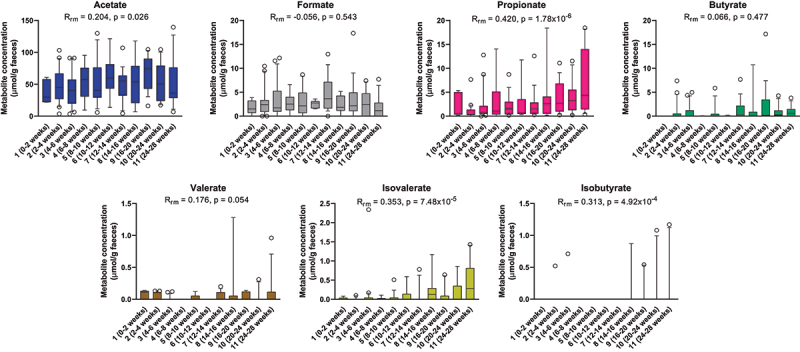
Longitudinal dynamics of short chain fatty acids in feces of infants in the CIG cohort. Statistical significance was evaluated based on repeated-measures correlations, with *R*_rm_ indicating the correlation coefficient between infant age and the indicated metabolite (*n*_infants_ = 25, *n*_datapoints_ = 144). Boxes and whiskers plot show median (black line), 25th–75th percentile (boxes) and 10th–90th percentile (whiskers).

We quantified the major SCFAs (acetate, butyrate, propionate, formate, and valerate) as well as the branched short-chain fatty acids (isobutyrate and isovalerate) in a subset of 144 stool samples across the 25 infants. Acetate was by far the most abundant SCFA, followed by formate and propionate. Butyrate, valerate, and isovalerate were less prevalent and detected in low quantities in most infants, whereas isobutyrate was only sporadically detected (Figure 2). A moderate increase with age was observed for acetate and valerate concentrations, whereas stronger increases in concentrations were seen for propionate, isovalerate, and isobutyrate, especially toward the last three sampling points (>20 weeks of age). By contrast, formate and butyrate concentrations did not change significantly over time within the first 6 months.

**Figure 3.**
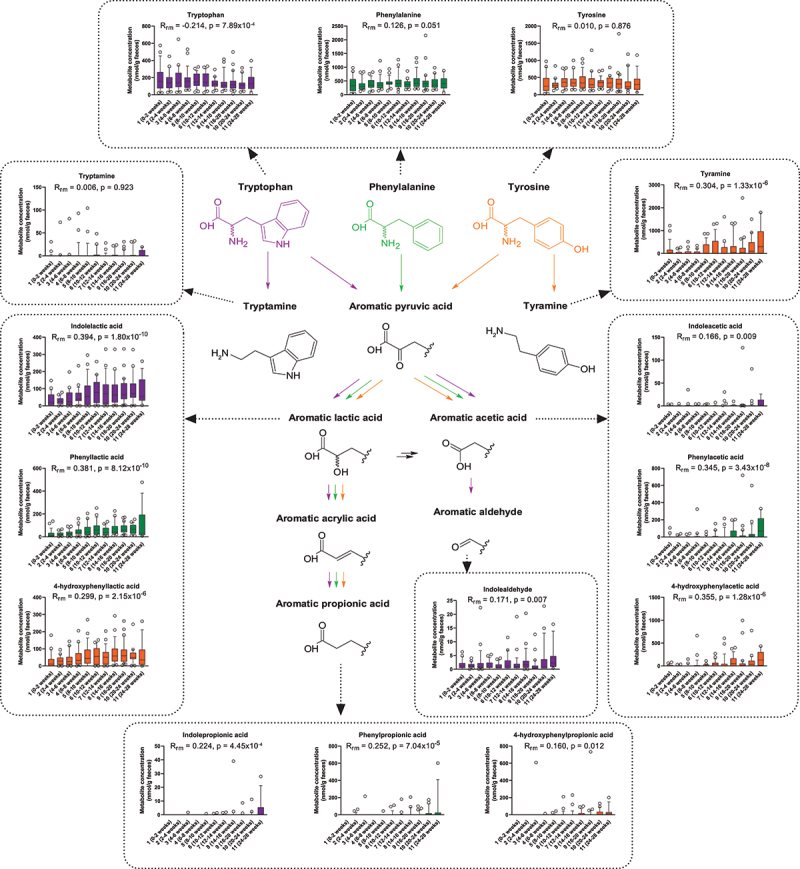
Longitudinal dynamics of AAAs and microbiota-derived catabolites in feces of infants in the CIG cohort. Statistical significance was evaluated based on repeated measures correlations, with *R*_rm_ indicating the correlation coefficient between infant age and the indicated metabolite concentration (*n*_infants_ = 25, *n*_datapoints_ = 267). Boxes and whiskers plot show median (black line), 25th–75th percentile (boxes) and 10th–90th percentile (whiskers). Plots of tryptophan, phenylalanine, and tyrosine metabolites are colored purple, green, and orange, respectively. Full color available online.

In contrast to SCFAs, knowledge about the temporal dynamics of the AAA catabolites in early life is extremely limited. Thus, we quantified the AAAs and their microbiota-derived catabolites in feces across all infants (Figure 3). Although less abundant than the SCFAs, some AAA catabolites were frequently detected. Apart from the AAAs, the aromatic lactic acids (4-hydroxyphenyllactic acid [OH-PLA], phenyllactic acid [PLA], and indolelactic acid [ILA]) were the most frequently detected metabolites found in the infants. Although the fecal abundance of the AAAs appeared more stable over time (even though a significant decrease in tryptophan was seen), many of the microbiota-derived AAA catabolites increased with age, however at different paces (Figure 3). Interestingly, the aromatic lactic acids were frequently detected even shortly after birth and steadily increased in feces until around 8–12 weeks of age where they plateaued. Conversely, the aromatic acetic acids (4-hydroxyphenylacetic acid [OH-PAA], phenylacetic acid [PAA], and indoleacetic acid [IAA]) were rarely detected until around 10–12 weeks of age where they started to increase toward the highest levels observed at 24–28 weeks of age. The aromatic propionic acids (4-hydroxyphenylpropionic acid [OH-PPA], phenylpropionic acid [PPA], and indolepropionic acid [IPA]) were rarely detected until the very last samplings at 20–28 weeks of age. The tyrosine-derived metabolite tyramine also showed a significant change with age, steadily increasing throughout the sampling period. This was, however not true for the tryptophan-derived metabolite tryptamine, which was detected throughout the sampling period without a consistent temporal pattern. Finally, indolealdehyde was, although in low abundance, frequently detected throughout the sampling period with a minor increase observed toward 24–28 weeks of age. When repeating our analyses using a subset of the cohort with only the exclusively breastfed infants (*n* = 13, all singletons, born vaginally at term) and excluding data points associated with solid foods or oral antibiotics, we observed similar patterns for both SCFAs and AAA catabolites versus infant age as for the whole cohort (Supplementary Table S1). Together these data confirm previous findings regarding SCFAs^19^ and demonstrate unprecedented early-life dynamics of the microbiota-derived AAA catabolites in breastfed infants.

### AAA catabolites associate with specific members of the infant gut microbiota

**Figure 4.**
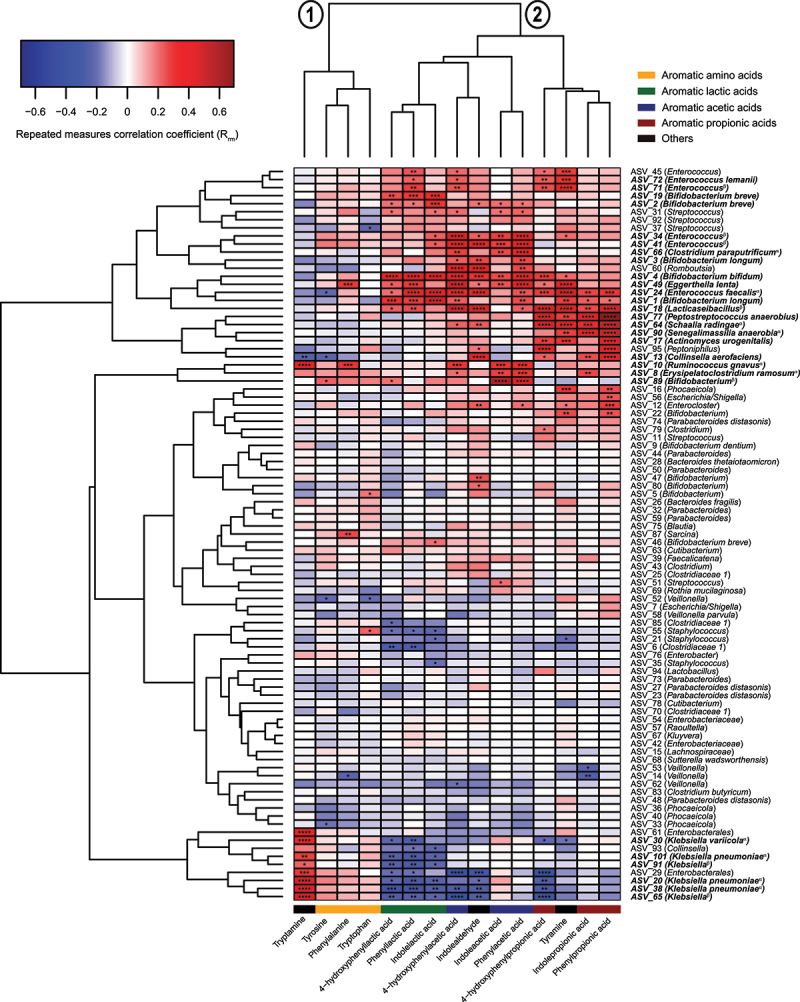
Bacterial taxa associate with the abundance of AAA catabolites in the CIG cohort. The heatmap shows hierarchical clustered repeated-measures correlation coefficients between the absolute abundance of the most abundant ASVs (average relative abundance > 0.05%) and the concentrations of the AAAs and their catabolites in feces from the CIG cohort. Cluster 1 contains the AAAs and tryptamine, whereas Cluster 2 contains the aromatic lactic acids, acetic acids, and propionic acids as well as indole aldehyde and tyramine. Statistical significance was evaluated by repeated-measures correlations with false discovery rate corrected *p*-values (*q*-values) shown by asterisks, * *q* < 0.05, ** *q *< 0.01, ***q < 0.001, and **** *q* < 0.0001. Bold face ASVs indicate taxa with significant association with the AAA catabolites that were selected for *in vitro* validation (see Fig. 5). Unless otherwise noted, ASV taxonomies were based on the RDP database. Superscript annotations for taxa indicate that α; annotation of species-level taxonomy was obtained based on BLAST analysis of the given ASV sequence against the 16S rRNA database at NCBI, and β; annotation of species-level taxonomy was not possible, since the ASV sequence matched multiple species within the given genus based on BLAST analysis against the 16S rRNA database at NCBI (see Supplementary Table S2).

Since key bacterial taxa producing the SCFAs in early life have previously been identified,^19,20^ we here focused on determining the key microbial species responsible for the production of AAA catabolites in infants’ gut. By performing repeated-measures correlation analyses between the absolute abundances of the most abundant ASVs (average relative abundance > 0.05%) and the AAA metabolites and hierarchical clustering of the coefficients, we found two main clusters (Figure 4). One cluster included the AAAs (and tryptamine), whereas the second cluster included the AAA catabolites. Within the second cluster, sub-clusters could be identified, largely representing the aromatic lactic acids, the aromatic acetic acids (and indolealdehyde), or the aromatic propionic acids (and tyramine). While a limited number of associations were found between ASV abundances and the abundances of the AAAs, multiple associations were found for all microbiota-derived AAA catabolites. Tryptamine correlated positively with ASV_10, matching *Ruminococcus gnavus*, as well as several ASVs belonging to *Klebsiella* species. As we have previously reported,^8^ the aromatic lactic acids were mainly associated with *Bifidobacterium* species, such as *B. bifidum*, *B. breve*, and *B. longum*, which all harbor an aromatic lactate dehydrogenase responsible for converting the aromatic pyruvic acids into the corresponding lactic acid forms. The aromatic acetic acids were linked to several different taxa including ASVs belonging to *Enterococcus*, *Bifidobacterium*, and ASV_18 (*Lacticaseilactobacillus*), ASV_8 (*Erysipelatoclostridium ramosum*), ASV_60 (*Clostridium paraputrificum*), and ASV_49 (*Eggerthella lenta*). Although the aromatic propionic acids and tyramine correlated with ASVs matching *Enterococcus* species, ASV_18 (*Lacticaseilactobacillus*), and ASV_13 (*Collinsella aerofaciens*), stronger associations were seen with ASV_64 (*Schaalia radingae*), ASV_90 (*Senegalimassilia anaerobia*), ASV_17 (*Actinomyces urogenitalis*), and, in particular, ASV_77 (*Peptostreptococcus anaerobious*). Together, these data suggest that common bacterial pathways across the three AAAs are used to produce specific groups of AAA catabolites and that particular taxa are responsible for the production of specific AAA catabolites in the infant gut.

### Bacterial taxa producing AAA catabolites *in vitro* show tight co-variation with these *in vivo*

**Figure 5.**
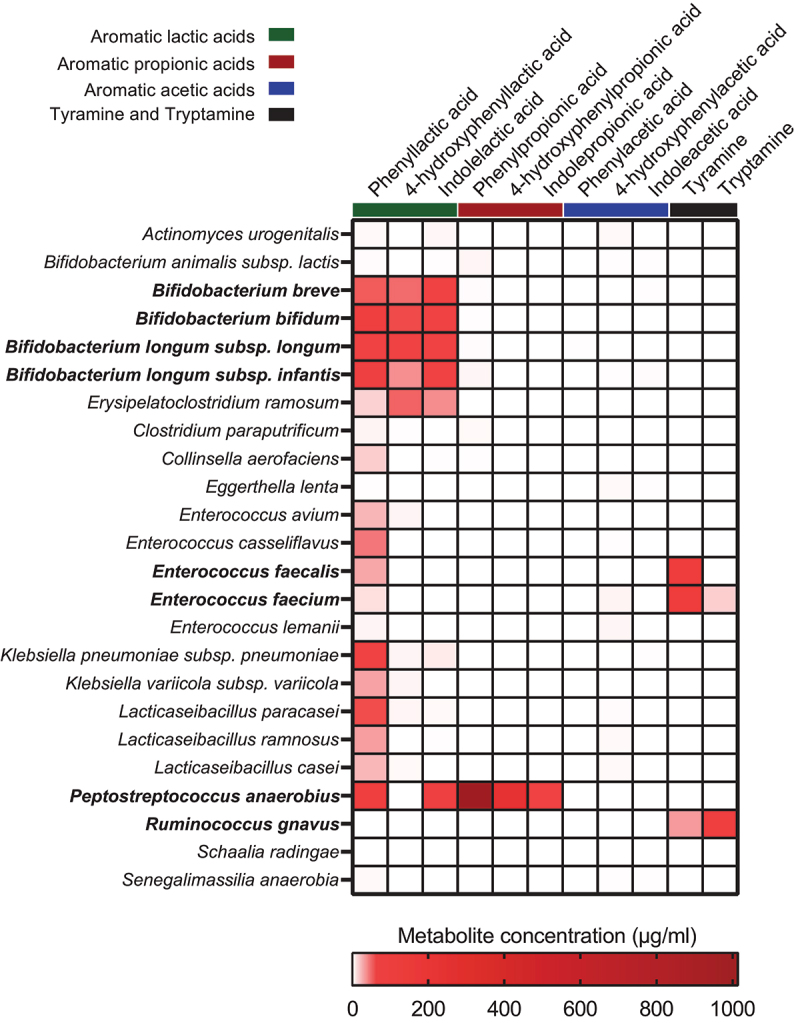
*In vitro* production of AAA catabolites by selected bacterial strains. Strains were cultured in BHI broth for 72 h at 37°C before assessing the amount of the AAA catabolites in the culture supernatants. Bold face text indicate the species where the main taxa–metabolite association from the CIG cohort was confirmed.

**Figure 6.**
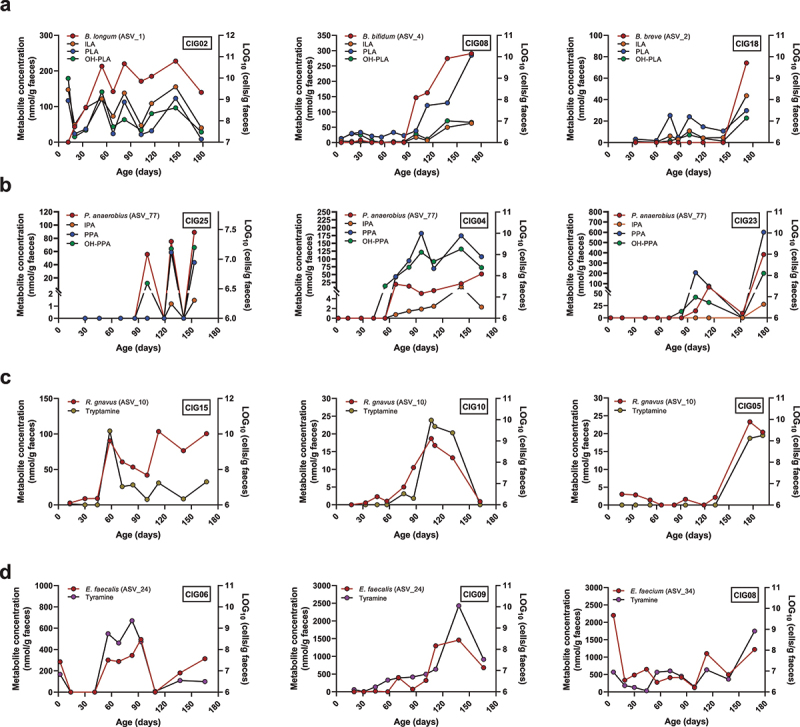
Intra-individual microbe–metabolite co-variation in feces of selected infants from the CIG cohort. (a) Examples of individuals showing co-variation between the abundance of aromatic lactic acids and *B. longum*, *B. bifidum*, or *B. breve*. (b) Examples of individuals showing co-variation between the abundance of the aromatic propionic acids and *P. anaerobius*. (c) Examples of individuals showing co-variation between the abundance of tryptamine and *R. gnavus*. (d) Examples of individuals showing co-variation between the abundance of tyramine and *E. faecalis* or *E. faecium.*

Having identified associations between specific taxa and abundances of individual AAA catabolites, we next sought to validate that the specific taxa indeed were able to produce the indicated AAA catabolites. We selected strains (Supplementary Table S2) representing the ASVs/species versus indicated AAA catabolites, based on the repeated-measures correlation analyses performed (Figure 4). We then mono-cultured representative strains within each associated species in the BHI broth and assessed the levels of the AAA catabolites in the culture supernatants following 72 h of growth (Figure 5). As we have previously demonstrated using the MRS broth,^8^ *Bifidobacterium* species harboring the *aldh* gene (*B. longum* subsp. *longum*, *B. longum* subsp. *infantis*, *B. breve*, and *B. bifidum*) were alsoable to produce the three aromatic lactic acids in the BHI broth, but none of the other measured metabolites. *B. animalis* subsp. *lactis*, a species lacking the *aldh*,^8^ did not produce significant amount of aromatic lactic acids nor any of the other measured metabolites. Importantly, we noted that PLA, but not the other aromatic lactic acids, was produced by a wide range of the tested species including *Enterococcus* spp. and *Lacticaseibacillus* spp., which also correlated with PLA in the infants. However, while PLA was also produced *in vitro* by *C. aerofaciens*, *Klebsiella* spp., and *E. ramosum*, these associations were not observed in infants. *P. anaerobius* was the only species found to produce the aromatic propionic acids in agreement with this being the taxon showing the strongest associations to these metabolites in infants (Figure 5). Although we found that *P. anaerobius in vitro* also produces aromatic lactic acids, no correlations were observed between these metabolites and *P. anaerobius* in the infants, which may reflect that the aromatic lactic acids are mainly intermediates in the pathway toward the final aromatic propionic acid products^13^ or simply that *Bifidobacterium* species are the main producers of the aromatic lactic acids during infancy. Despite the fact that multiple taxa showed significant positive correlations to the aromatic acetic acids, we failed to detect these metabolites *in vitro* for the associated species (e.g. *E. faecalis*, *Lacticaseibacillus* spp., *S. radingae*, *S. anaerobia*, *A. urogenitalis*, and *C. aerofaciens*). Multiple taxa correlated with tyramine in the CIG infants. Yet, tyramine was only measured in the culture supernatants of *E. faecalis* and *E. faecium* (but not the other *Enterococcus* spp. tested). Tyramine was also produced in lower quantities by *R. gnavus*, but this taxon did not show significant correlation to tyramine in the infants. With the exception of trace amounts found in the culture supernatant of *E. faecium*, tryptamine was solely produced *in vitro* by *R. gnavus* and not measured in any of the culture supernatants from the *Klebsiella* species tested. Thus, our *in vitro* experiments show that some, but not all, metabolite–microbe associations observed in infants could be validated. To further illustrate the validated microbe-metabolite relations, we examined intra-individual co-variation of the absolute abundance of relevant species and metabolites in selected participants from the CIG cohort (Figure 6). This showed that distinct *aldh* encoding *Bifidobacterium* species^8^ exhibited clear covariation with the aromatic lactic acids in different individuals, colonized by *B. longum*, *B. bifidum*, or *B. breve* (Figure 6a). Furthermore, *P. anaerobius* showed co-variation over time with the aromatic propionic acids in different individuals (Figure 6b), *R. gnavus* strongly co-varied over time with tryptamine concentration in multiple infants (Figure 6c), and *E. faecalis* or *E. faecium* co-varied with tyramine concentrations over time depending on the infant (Figure 6d). Taken together, we present evidence suggesting that these aforementioned species are key producers of AAA catabolites in infants’ gut in the first 6 months of life.

## Discussion

In a cohort of 25 Danish infants, we assessed the development of the gut microbiota and metabolome. In line with previous studies^19,20^ we found that age affects the diversity and composition of the gut microbiota but also found that subject ID was by far a stronger determinant, highlighting the individuality of the gut microbiota even across infants who are all primarily breastfed. Furthermore, moving beyond the study of the microbiota, we benefited from the longitudinal study design and demonstrated dynamics of the SCFAs in feces in agreement with previous studies.^19,21^ The initial increase in acetate happens within the first few months of life then stagnates, followed by an increase in propionate occurring steadily during the first 6 months of age, and butyrate being less abundant and prevalent during the first 6 months of age. The lack of a significant increase in butyrate is likely due to the absence of typical major butyrate producers (*Clostridiales* species such as *Faecalibacterium prausnitzii*) that often colonize during the last 6 months of infancy,^21,22^ which is beyond our sampling regime. We observed limited, but increasing, valerate, isobutyrate, and isovalerate concentrations during the first 6 months of life, which is in line with previous studies showing that these are produced in much higher quantities toward the end of the first year of life.^19^

Extending previous findings, our results demonstrate hitherto unprecedented longitudinal dynamics of key microbiota-derived AAA catabolites in infants’ gut. Importantly, while the AAAs were rather stable over time or decreased slightly (in the case of tryptophan), a temporal increase was observed for most of the microbiota-derived AAA catabolic products. Specifically, we observed an initial increase in the aromatic lactic acids from the very first samplings oc curring at 0–2 weeks of age until 10–12 weeks of age, followed by the appearance and increase of the aromatic acetic acids around 10–12 weeks of age and the later appearance and increase in the aromatic propionic acid detected only in a few infants before 20–28 weeks of age.

In our efforts to link specific gut bacterial taxa and AAA catabolism, we confirmed *in vitro* production of the aromatic lactic acids by *aldh* encoding *Bifidobacterium* species,^8^ aromatic propionic acids production by *P. anaerobius*, tyramine production by *E. faecalis* and *E. faecium* as well as tryptamine production by *R. gnavus*, which is completely consistent with the genetic and phenotypic evidence of these species to produce these metabolites in prior studies.^8,13,23^ In addition, PLA production was confirmed in all *Enterococcus* and *Lacticaseibacillus* species tested, in agreement with previous reports on the production of this metabolite from species within in these genera.^24,25^ Notably, for 10 out of the 24 strains that we tested *in vitro* we did not detect any of the AAA catabolites that were expected based on the *in vivo* associations. Although we cannot rule out that AAA catabolite production in some cases may depend on interspecies cross-feeding and/or the specific *in vitro* culture conditions, this highlights that correlation does not always mean causation, emphasizing that it remains essential, by all means possible, to validate findings of correlative nature. Remarkably, we were not able to validate any producers of the aromatic acetic acids *in vitro*, despite correlations to multiple different bacterial taxa in the infants. However, in order to exclude the possibility that the lack of detecting the aromatic acetic acids was due to poor production in the medium or technical issues of measuring the metabolites, we also cultured *Bacteroides thetaiotaomicron*, a known producer of PAA and OH-PAA,^26^ and detected both of these compounds in the culture supernatant (Supplementary Figure S1). Surprisingly, even though we did detect an ASV with an exact match to *B. thetaiotaomicron* (ASV_28) in the CIG infants, it did not associate with the presence/abundance of PAA and OH-PAA, suggesting that it may not be a key producer of these metabolites in the infant gut. Therefore, it remains unknown which microbial species are responsible for the production of the aromatic acetic acids in infants’ gut.

The identification of key producers of AAA catabolites were corroborated by the observed longitudinal microbe–metabolite co-variation within the infants. This illustrates not only how a single bacterial species within an individual in the context of early infancy can determine the production of a specific AAA catabolite (Figure 6b, c) but also that different species can be responsible for the production of the same AAA catabolite in different individuals (Figure 6a, c). We have previously elucidated^8^ that the initial appearance and increase in aromatic lactic acids arise because of the initial increase in HMO-utilizing *Bifidobacterium* species harboring the aromatic lactate dehydrogenase encoding gene (*aldh*) enabling them to convert the AAAs into aromatic lactic acids via the aromatic pyruvic acid intermediates. Indeed, this is diet-dependent, as several studies have demonstrated that fecal abundance of the aromatic lactic acids is markedly reduced in formula compared to breastfed infants,^27–29^ and in weaned compared to partially breastfed infants,^8^ most likely due to the reduction or complete absence of HMO-utilizing *Bifidobacterium* species in formula-fed and weaned infants. However, due to the very high breastfeeding prevalence, limited formula use, and limited exposure to solid foods in our cohort, we were not able to assess the effects of diet on gut microbial metabolism. The low sample size also hindered us to study the impact of antibiotics, mode of delivery, and gestational age, which are likely to be important factors as well that should be also assessed in future studies. In addition, metabolite concentrations were estimated based on per g of wet stool and not normalized to dry weight due to limited feces material being available. Since stool consistency may change during infancy,^30^ the metabolite concentrations reported here may partly be affected by the water content of stool. We did, however, not observe major consistent differences in stool consistency over the course of the sampling period, likely reflecting the limited amount of solid foods introduced within our sampling frame. Another limitation of our study is that stool concentrations of the measured metabolites will not fully reflect intestinal concentrations due to absorption in the gut as well as potential degradation and evaporation from defecation until analysis. In this study, parents were instructed to place the sampled stool in the home freezer immediately after fresh collection from the nappy, before transportation on dry ice and storage at −80°C and a single defrosting following immediate processing, limiting potential issues with evaporation, and degradation. Although the reported concentrations should be interpreted with care, this does not compromise our conclusions on the relative changes observed longitudinally nor does it influence the demonstrated microbe–metabolite associations from these stool samples. Indeed, our study provides novel insights into the dynamics of two important classes of microbiota-derived metabolites during infancy. The longitudinal infant cohort approach combined with *in vitro* cultivation of strains for the validation of microbe–metabolite associations is a strong framework, which could be applied in future infant studies with frequent sampling and omics analyses.

The temporal changes in fecal concentrations of many of the AAA catabolites observed here may indeed have consequences for the current and future health of infants.^6^ For example, several recent studies have reported the beneficial effects of aromatic lactic acids, in particular ILA, for early life immune development.^8,31–33^ Indole propionic acid, a metabolite observed only in some infants and mainly in the last samplings, has been shown to decrease the permeability of the gut epithelium,^13^ exhibit neuroprotective activity,^18^ and is inversely linked to the risk of developing type 2 diabetes.^34,35^ However, further research is required to fully understand the potential health implications of the different AAA catabolites and their temporal changes in the early life context.

In summary, we described the temporal dynamics of SCFAs and AAA catabolites in the infant gut during the first 6 months of life and demonstrated that specific AAA catabolites result from the appearance and abundance of specific key bacterial taxa. Our findings provide key directions for future mechanistic work on gut microbial metabolism in the infant gut, which should be validated and extended beyond the first 6 months of life. Such studies are needed as knowledge about the developmental pattern of microbiota-derived metabolites is essential for advancing our understanding of diet–microbe–host interactions in early life, which is pivotal for the realization of gut-directed strategies supporting and nurturing healthy development during infancy.

## Methods

### Copenhagen Infant Gut cohort: sample collection and metadata

The Copenhagen Infant Gut (CIG) cohort consisted of 25 healthy infants (13 females and 12 males) from whom fecal samples were obtained throughout the first 6 months of age, at 11 distinct time points (0–2 weeks, 2–4 weeks, 4–6 weeks, 6–8 weeks, 8–10 weeks, 10–12 weeks, 12–14 weeks, 14–16 weeks, and 16–20 weeks, 20–24 weeks and 24–28 weeks of age) as previously described^8^. In brief, parents collected fresh fecal samples from nappies into sterile feces collection tubes (Sarstedt) and immediately stored them at −18°C in a home freezer until transportation to the Technical University of Denmark where the samples were stored at −80°C until sample preparation. Parents filled in a sheet with information about their child’s gender, term and mode of delivery, consumption of antibiotics, milk feeding patterns, and timing of introduction to solid foods (Table 1). No health measurements of the infants were obtained. The Data Protection Agency (18/02459) approved the study and the office of the Committees on Biomedical Research Ethics for the Capital Region of Denmark confirmed that the study was not notifiable according to the Act on Research Ethics Review of Health Research Projects (paragraph 1, subsection 4). Informed consent was obtained from all parents of infants participating in the study. In addition, the parents of the twins gave informed consent to publish data from the twins although the parents themselves would be able to identify their children using indirect identifiers. The parents did not receive any compensation.

### 16S rRNA gene amplicon sequencing

As previously published,^8^ we extracted DNA and 16S rRNA gene amplicon sequenced 269 fecal samples from the 25 infants participating in the CIG study. However, data from a total of 34 samples were missing/excluded due to insufficient sample material (*n* = 1), insufficient DNA extraction/lack of PCR product (*n* = 20), a low number of sequencing reads (*n* = 6) or resemblance of community to sequenced blank buffer DNA extraction negative controls (*n* = 1), or too low sequencing depth for diversity analyses (*n* = 6), ending up with 235 samples. Briefly, DNA was extracted from 250 mg of feces or blank buffer negative controls (PowerLyzer® PowerSoil® DNA isolation kit, MoBio 12,855–100) and the V3 region of the 16S rRNA gene was amplified (30 s at 98°C, 30 cycles of 15 s at 98°C and 30s at 72°C, followed by 5 min at 72°C) using non-degenerate universal barcoded primers^36^ and then sequenced with the Ion OneTouch^TM^ and Ion PGM platform with a 318-Chip v2. Sequences were de-multiplexed according to barcode and trimmed as previously described^36^ in CLC Genomic Workbench (v8.5. CLCbio, Qiagen, Aarhus, DK). The resulting sequences were then analyzed using the DADA2 pipeline^37^ (v. 1.14) by quality filtering (maxEE = 1), learning errors, and denoising (pool=TRUE, HOMOPOLYMER_GAPPENALTY = −1 and BAND_SIZE = 32) as recommended for Ion Torrent reads, and chimera filtration, with otherwise default settings. The resulting 806 Amplicon Sequence Variants (ASVs) were assigned taxonomy using the RDP classifier^38^ and RDP 16S rRNA database (v.18). Additionally, for selected ASV, taxonomy was confirmed by BLAST analyses of these sequences against the 16S rRNA database at NCBI. Using QIIME2^39^, ASVs assigned to cyanobacteria/chloroplast or with a frequency <100 reads across all samples were removed, ending up with 343 ASVs. Based on these, the *core diversity metrics* function was applied with a rarefaction depth of 8,000 reads per sample to generate weighted and unweighted UniFrac distance matrices and principal coordinates, as well as alpha diversity measures (Shannon index, Observed ASVs, and Evenness index). Using the *rarefy table* function, the ASV table was rarefied to 8,000 reads per sample and collapsed to higher taxonomical levels using the *taxa collapse* function. Relative abundances at all levels were calculated by the total sum scaling, and absolute abundances for each taxon were estimated by multiplying relative abundance with qPCR-estimated total bacterial load (see below), however with a minimum load set to 10^6^ cells/g feces for features below the detection limit of 16S rRNA amplicon sequencing.

### Quantitative PCR

Using universal primers (341F: 5ʹ-CCTACGGGAGGCAGCAG-3ʹ, 518 R: 5ʹ-ATTACCGCGGCTGCTGG-3ʹ, with final concentration of 0.2 µM each) the total bacterial load was estimated by quantitative PCR, as previously published.^8^ Each reaction was performed (in triplicates) with 5 µl PCR-grade water, 1.5 µl forward and reverse primer, 10 µl SYBR Green I Master 2× (LightCycler® 480 SYBR Green I Master, Roche, 04887352001), and 2 µl template DNA, in a total volume of 20 µl. Standard curves were generated from 10-fold serial dilutions of linearized plasmid (containing 10^8^−10^0^ gene copies/ul), constructed by cloning a PCR amplified 199bp fragment of the 16S rRNA gene (V3-region) of *E. coli* (ATCC 25922) into a pCR4-Blunt-TOPO vector (Invitrogen). Plates were run on the LightCycler® 480 Instrument II (Roche, 05015243001) with the program including 5 min pre-incubation at 95°C, followed by 45 cycles with 10 sec at 95°C, 15 sec at 60°C, and 15 sec at 72°C and a subsequent melting curve analysis including 5 min at 95°C, 1 min at 65°C and continuous temperature increase (ramp rate 0.11°C/s) until 98°C. Data were analyzed with the LightCycler® 480 Software (v 1.5). Bacterial load data were used to estimate the absolute abundances of each microbial taxa by multiplying with relative abundances (derived from 16S rRNA gene amplicon sequencing).

### Bacteria and culture conditions

All the representative bacterial strains listed in Supplementary Table 2 were purchased from DSMZ (German Collection of Microorganisms and Cell Cultures GmbH, Germany). They were revived on BHI (Brain heart infusion) plates, glycerol stocks were prepared and stored at −80°C until further use. BHI media were always supplemented with hemin (5 µg/ml), vitamin K (50 µg/ml), and cysteine (0.5 mg/ml). For culturing experiments, bacterial strains were revived on BHI plates and grown overnight as primary cultures in BHI medium under mild shaking conditions. The next morning, they were then diluted to OD_600_ = 0.02 in 3 ml BHI medium as secondary cultures and grown for 72 hrs in mild shaking conditions. Each strain was cultured at least in triplicates. Culture media without inoculation were used as controls. After 72 hrs of fermentation, OD_600_ was measured and samples were put on ice. About 1 ml each of the samples was centrifuged at 14,000 rpm for 10 minutes at 4°C, and the supernatants were collected and stored at −20°C. They were processed for metabolite extraction and analysis as described in the next section below. All growth experiments were performed inside the Whitley A95 anaerobic workstation maintained at 37°C and all the plates and media were incubated inside the workstation at least 24 hrs before use to maintain anoxic conditions.

### Metabolite extraction and profiling of samples

#### Chemicals

Authentic standards of the AAAs and derivatives were obtained from Sigma Aldrich, whereas isotope-labeled AAAs used as internal standards (L-Phenylalanine (ring-d5, 98%), L-Tyrosine (ring-d4, 98%), L-Tryptophan (indole-d5, 98%) and indoleacetic acid (2,2-d2, 96%)) of the highest purity grade available were obtained from Cambridge Isotope Laboratories Inc.

#### Quantification of short-chain fatty acid in fecal samples

Short-chain fatty acids were quantified in approximately every second fecal sample obtained from each individual (*n* = 144) from approximately 150 mg of fecal matter by MS-Omics ApS (Vedbæk, Denmark) as follows. Samples were mixed with ultrapure water (2 µL/mg) and ultrasonicated for 10 minutes. Hereafter samples were acidified using hydrochloric acid fortified with stable isotope-labeled internal standards and centrifuged (20 min at 16,000 g). The supernatants were then transferred to GC-MS vials for analysis. All samples were analyzed in a randomized order. Analysis was performed using a high polarity column (Zebron^TM^ ZB-FFAP, GC Cap. Column 30 m x 0.25 mm x 0.25 µm) installed in a GC (6890N, Agilent) coupled with a quadrupole detector (5975B, Agilent). Raw data were converted to netCDF format using ChemStation before the data were imported and processed in Matlab R2014b (MathWorks, Inc.) using the PARADISe software described by Johnsen *et al*^40^.

#### Extraction of metabolites from fecal samples for AAA metabolite profiling

The extraction of AAA metabolites from fecal samples has previously been described.^8^ In brief, except 2 samples with insufficient amounts of material, metabolites were extracted from the remaining 267 fecal samples (100–500 mg) using sterile milli-Q water, the addition of internal standards, protein precipitations by acetonitrile, drying by nitrogen gas followed by reconstitution of residues in milli-Q water resulting in a final dilution of 1:5 of the fecal sample.

#### *Extraction of metabolites from* in vitro *fermentation samples for AAA metabolite profiling*

As described previously,^8^ culture supernatants from *in vitro* fermentations were thawed at 4°C and then centrifuged at 16,000×g at 4°C for 10 minutes. Subsequently, 80 µL was transferred to a new tube and 20 µL internal standard (40 µg/mL) plus 300 µL acetonitrile were added. These samples were vortexed for 10 seconds and left at −20°C for 10 minutes in order to precipitate the proteins. Then, samples were centrifuged at 16,000×g, 4°C for 10 minutes before 50 µL supernatant of each sample was diluted with 50 µL of sterile water and transferred to a liquid chromatography vial (equaling a 1:10 dilution of the sample with internal standards having a concentration of 1 µg/mL).

#### *AAA metabolite profiling of fecal and* in vitro *samples*

AAAs and catabolites were semi-quantified in fecal and *in vitro* samples by ultra performance liquid chromatography mass spectrometry (UPLC-MS) using isotopic internal standards with similar molecular structures as previously published.^8^ In brief, the samples (2 µL of each) were analyzed in random order, however with all samples of the same individual analyzed on the same day by a quadrupole time-of-flight mass spectrometry (UPLC-QTOF-MS) system consisting of Dionex Ultimate 3000 RS liquid chromatograph (Thermo Scientific) coupled to a Bruker maXis time of flight mass spectrometer equipped with an electrospray interphase (Bruker Daltonics) operating in positive mode. The analytes were separated on a Poroshell 120 SB-C18 column with a dimension of 2.1 × 100 mm and 2.7 μm particle size (Agilent Technologies) as previously published.^8^ For analysis of fecal samples, a pooled quality control (QC) sample was injected for every 10 samples to monitor the system stability and standard mix solutions (0.1 μg/mL, 0.5 μg/mL, 1 μg/mL, 2 μg/mL, and 4 μg/mL) were analyzed once for every 10 samples to obtain a standard curve for every 10 samples. Data were processed using QuantAnalysis version 2.2 (Bruker Daltonics) and bracket calibration curves (fitted to a quadratic regression) for every 10 fecal samples were obtained for each metabolite. For *in vitro* samples, another layer of QC was done by taking standard mix solutions of all the analytes (2 µg/mL) in the culture medium and processed similar to the culture supernatant samples to normalize against any loss of the analytes during the processing. In addition, QC samples and standard mix solutions were analyzed before and after all the samples and after every 10 samples two standards were analyzed and data were processed using QuantAnalysis version 2.2 (Bruker Daltonics) and a calibration curve (fitted to a quadratic regression) with all standards analyzed for each metabolite. The calibration curves were established by plotting the peak area ratios of all of the analytes with respect to the internal standard against the concentrations of the calibration standards.

## Statistics

Statistical analyses were performed in R (v3.6.1). In order to assess the effect of different variables on gut microbiota composition PERMANOVA implemented in the *adonis.2* function (*n*_permutation_ = 999) within R package *vegan* (v2.5–7) was used with weighted or unweighted UniFrac distances as input, with permutations restricted within individuals (strata=ID) when assessing the effect of age. To investigate the temporal dynamics of gut microbial alpha diversity as well as temporal changes in amounts of AAA metabolites and SCFAs, repeated measures correlation analyses were performed in R using the *rmcorr* (v0.4.3) package. Further, repeated measures correlation analyses were used to look for associations between Log_10_ transformed absolute abundance of ASVs (>0.05% average relative abundance) and abundance of AAA metabolites, illustrated by a heatmap with hierarchical clustering of the correlation coefficient, using the *heatmap.2* function within the *gplots* (v3.1.1) package. The resulting *p*-values were corrected for multiple testing by the Benjamini – Hochberg false discovery rate (FDR) using a cutoff of 0.05, yielding *q*-values. R codes are available upon request.

## Supplementary Material

Supplemental Material

## Data Availability

16S rRNA gene amplicon sequencing data has been deposited in the Sequence Read Archive (SRA) under BioProject PRJNA554596 (CIG).

## References

[1] Tamburini S, Shen N, Wu HC, Clemente JC. The microbiome in early life: implications for health outcomes. Nat Med. 2016;22(7):713–17. doi:10.1038/nm.4142. 27387886

[2] Dominguez-Bello MG, Godoy-Vitorino F, Knight R, Blaser MJ. Role of the microbiome in human development. Gut. 2019;68(6):1108–1114. doi:10.1136/gutjnl-2018-317503. 30670574 PMC6580755

[3] Laue HE, Coker MO, Madan JC. The developing microbiome from birth to 3 years: the gut-brain axis and neurodevelopmental outcomes. Front Pediatr. 2022;10:254. doi:10.3389/fped.2022.815885. PMC8936143 35321011

[4] Jain N, Walker WA. Diet and host–microbial crosstalk in postnatal intestinal immune homeostasis. Nat Rev Gastroenterol Hepatol. 2015;12(1):14–25. doi:10.1038/nrgastro.2014.153. 25201040 PMC11453094

[5] Roager HM, Dragsted LO. Diet‐derived microbial metabolites in health and disease. Nutr Bull. 2019;44(3):216–227. doi:10.1111/nbu.12396.

[6] Roager HM, Stanton C, Hall LJ. Microbial metabolites as modulators of the infant gut microbiome and host-microbial interactions in early life. Gut Microbes. 2023;15(1):15. doi:10.1080/1949097620232192151. PMC10038037 36942883

[7] Koh A, De Vadder F, Kovatcheva-Datchary P, Bäckhed F. From dietary fiber to host physiology: short-chain fatty acids as key bacterial metabolites. Cell. 2016;165(6):1332–1345. Cell2016. doi:10.1016/j.cell.2016.05.041. 27259147

[8] Laursen MF, Sakanaka M, von Burg N, Mörbe U, Andersen D, Moll JM, Pekmez CT, Rivollier A, Michaelsen KF, Mølgaard C, et al. *Bifidobacterium* species associated with breastfeeding produce aromatic lactic acids in the infant gut. Nat Microbiol. 2021;6(11):1367–1382. doi:10.1038/s41564-021-00970-4. 34675385 PMC8556157

[9] Roager HM, Licht TR. Microbial tryptophan catabolites in health and disease. Nat Commun. 2018;9(1):3294. doi:10.1038/s41467-018-05470-4. 30120222 PMC6098093

[10] Natividad JM, Agus A, Planchais J, Lamas B, Jarry AC, Martin R, Michel ML, Chong-Nguyen C, Roussel R, Straube M, et al. Impaired aryl hydrocarbon receptor ligand production by the gut microbiota is a key factor in metabolic syndrome. Cell Metab. 2018;28(5):737–749.e4. doi:10.1016/j.cmet.2018.07.001. 30057068

[11] Hoyles L, Fernández-Real J-M, Federici M, Serino M, Abbott J, Charpentier J, Heymes C, Luque JL, Anthony E, Barton RH, et al. Molecular phenomics and metagenomics of hepatic steatosis in non-diabetic obese women. Nat Med. 2018;24(7):1070–1080. doi:10.1038/s41591-018-0061-3. 29942096 PMC6140997

[12] Krishnan S, Ding Y, Saedi N, Choi M, Sridharan GV, Sherr DH, Yarmush ML, Alaniz RC, Jayaraman A, Lee K. Gut microbiota-derived tryptophan metabolites modulate inflammatory response in hepatocytes and macrophages. Cell Rep. 2018;23(4):1099–1111. doi:10.1016/j.celrep.2018.03.109. 29694888 PMC6392449

[13] Dodd D, Spitzer MH, Van Treuren W, Merrill BD, Hryckowian AJ, Higginbottom SK, Le A, Cowan TM, Nolan GP, Fischbach MA, et al. A gut bacterial pathway metabolizes aromatic amino acids into nine circulating metabolites. Nature. 2017;551(7682):648–652. doi:10.1038/nature24661. 29168502 PMC5850949

[14] Zelante T, Iannitti RG, Cunha C, DeLuca A, Giovannini G, Pieraccini G, Zecchi R, D’Angelo C, Massi-Benedetti C, Fallarino F, et al. Tryptophan catabolites from microbiota engage aryl hydrocarbon receptor and balance mucosal reactivity via interleukin-22. Immunity. 2013;39(2):372–385. doi:10.1016/j.immuni.2013.08.003. 23973224

[15] Guo X, Liang Y, Zhang Y, Lasorella A, Kee BL, Fu Y-X. Innate lymphoid cells control early colonization resistance against intestinal pathogens through ID2-dependent regulation of the microbiota. Immunity. 2015;42(4):731–743. doi:10.1016/j.immuni.2015.03.012. 25902484 PMC4725053

[16] Hwang IK, Yoo KY, Li H, Park OK, Lee CH, Choi JH, Jeong YG, Lee YL, Kim YM, Kwon YG, et al. Indole-3-propionic acid attenuates neuronal damage and oxidative stress in the ischemic hippocampus. J Neurosci Res. 2009;87(9):2126–2137. doi:10.1002/jnr.22030. 19235887

[17] Mimori S, Kawada K, Saito R, Takahashi M, Mizoi K, Okuma Y, Hosokawa M, Kanzaki T. Indole-3-propionic acid has chemical chaperone activity and suppresses endoplasmic reticulum stress-induced neuronal cell death. Biochem Biophys Res Commun. 2019;517(4):623–628. doi:10.1016/j.bbrc.2019.07.074. 31378367

[18] Serger E, Luengo-Gutierrez L, Chadwick JS, Kong G, Zhou L, Crawford G, Danzi MC, Myridakis A, Brandis A, Bello AT, et al. The gut metabolite indole-3 propionate promotes nerve regeneration and repair. Nature. 2022;607(7919):585–592. doi:10.1038/s41586-022-04884-x. 35732737

[19] Tsukuda N, Yahagi K, Hara T, Watanabe Y, Matsumoto H, Mori H, Higashi K, Tsuji H, Matsumoto S, Kurokawa K, et al. Key bacterial taxa and metabolic pathways affecting gut short-chain fatty acid profiles in early life. Isme J. 2021;15(9):2574–2590. doi:10.1038/s41396-021-00937-7. 33723382 PMC8397723

[20] Yatsunenko T, Rey FE, Manary MJ, Trehan I, Dominguez-Bello MG, Contreras M, Magris M, Hidalgo G, Baldassano RN, Anokhin AP, et al. Human gut microbiome viewed across age and geography. Nature. 2012;486(7402):222–227. doi:10.1038/nature11053. 22699611 PMC3376388

[21] Appert O, Garcia AR, Frei R, Roduit C, Constancias F, Neuzil-Bunesova V, Ferstl R, Zhang J, Akdis C, Lauener R, et al. Initial butyrate producers during infant gut microbiota development are endospore formers. Environ Microbiol. 2020;22(9):3909–3921. doi:10.1111/1462-2920.15167. 32686173

[22] Laursen MF, Laursen RP, Larnkjær A, Mølgaard C, Michaelsen KF, Frøkiær H, Bahl MI, Licht TR, Suen G. Faecalibacterium gut colonization is accelerated by presence of older siblings. mSphere. 2017;2(6):e00448–17. doi:10.1128/mSphere.00448-17. 29202044 PMC5705805

[23] Sugiyama Y, Mori Y, Nara M, Kotani Y, Nagai E, Kawada H, Kitamura M, Hirano R, Shimokawa H, Nakagawa A, et al. Gut bacterial aromatic amine production: aromatic amino acid decarboxylase and its effects on peripheral serotonin production. Gut Microbes. 2022;14(1):2128605. doi:10.1080/19490976.2022.2128605. 36217238 PMC9553188

[24] Valerio F, Lavermicocca P, Pascale M, Visconti A. Production of phenyllactic acid by lactic acid bacteria: an approach to the selection of strains contributing to food quality and preservation. FEMS Microbiol Lett. 2004;233(2):289–295. doi:10.1111/j.1574-6968.2004.tb09494.x. 15063498

[25] Ohhiral I, Kuwaki S, Morita H, Suzuki T, Tomita S, Hisamatsu S, Sonoki S, Shinoda S. Identification of 3-phenyllactic acid as a possible antibacterial substance produced by enterococcus faecalis TH 10. Biocontrol Sci. 2004;9(3):77–81. doi:10.4265/bio.9.77.

[26] Russell WR, Duncan SH, Scobbie L, Duncan G, Cantlay L, Calder AG, Anderson SE, Flint HJ. Major phenylpropanoid-derived metabolites in the human gut can arise from microbial fermentation of protein. Mol Nutr Food Res. 2013;57(3):523–535. doi:10.1002/mnfr.201200594. 23349065

[27] Sillner N, Walker A, Lucio M, Maier TV, Bazanella M, Rychlik M, Haller D, Schmitt-Kopplin P. Longitudinal profiles of dietary and microbial metabolites in formula- and breastfed infants. Front Mol Biosci. 2021;8:490. doi:10.3389/fmolb.2021.660456. PMC8195334 34124150

[28] Chow J, Panasevich MR, Alexander D, Vester Boler BM, Rossoni Serao MC, Faber TA, Bauer LL, Fahey GC. Fecal metabolomics of healthy breast-fed versus formula-fed infants before and during in vitro batch culture fermentation. J Proteome Res. 2014;13(5):2534–2542. doi:10.1021/pr500011w. 24628373

[29] He X, Parenti M, Grip T, Lönnerdal B, Timby N, Domellöf M, Hernell O, Slupsky CM. Fecal microbiome and metabolome of infants fed bovine MFGM supplemented formula or standard formula with breast-fed infants as reference: a randomized controlled trial. Sci Rep. 2019;9(1):1–14. doi:10.1038/s41598-019-48858-y. 31406230 PMC6690946

[30] Béghin L, Marchandise X, Lien E, Bricout M, Bernet JP, Lienhardt JF, Jeannerot F, Menet V, Requillart JC, Marx J, et al. Growth, stool consistency and bone mineral content in healthy term infants fed sn-2-palmitate-enriched starter infant formula: a randomized, double-blind, multicentre clinical trial. Clin Nutr. 2019;38(3):1023–1030. doi:10.1016/j.clnu.2018.05.015. 29903473

[31] Ehrlich AM, Pacheco AR, Henrick BM, Taft D, Xu G, Huda MN, Mishchuk D, Goodson ML, Slupsky C, Barile D, et al. Indole-3-lactic acid associated with *Bifidobacterium*-dominated microbiota significantly decreases inflammation in intestinal epithelial cells. BMC Microbiol. 2020;20(1):357. doi:10.1186/s12866-020-02023-y. 33225894 PMC7681996

[32] Henrick BM, Rodriguez L, Lakshmikanth T, Pou C, Henckel E, Arzoomand A, Olin A, Wang J, Mikes J, Tan Z, et al. Bifidobacteria-mediated immune system imprinting early in life. Cell. 2021;184(15):3884–3898.e11. doi:10.1016/j.cell.2021.05.030. 34143954

[33] Meng D, Sommella E, Salviati E, Campiglia P, Ganguli K, Djebali K, Zhu W, Walker WA. Indole-3-lactic acid, a metabolite of tryptophan, secreted by *Bifidobacterium longum* subspecies infantis is anti-inflammatory in the immature intestine. Pediatr Res. 2020;88(2):209–217. doi:10.1038/s41390-019-0740-x. 31945773 PMC7363505

[34] De Mello VD, Paananen J, Lindström J, Lankinen MA, Shi L, Kuusisto J, Pihlajamäki J, Auriola S, Lehtonen M, Rolandsson O, et al. Indolepropionic acid and novel lipid metabolites are associated with a lower risk of type 2 diabetes in the Finnish Diabetes Prevention Study. Sci Reports. 2017;7(1):1–12. 71 2017. doi:10.1038/srep46337. PMC5387722 28397877

[35] Tuomainen M, Lindström J, Lehtonen M, Auriola S, Pihlajamäki J, Peltonen M, Tuomilehto J, Uusitupa M, De Mello VD, Hanhineva K. Associations of serum indolepropionic acid, a gut microbiota metabolite, with type 2 diabetes and low-grade inflammation in high-risk individuals. Nutr Diabetes. 2018;8(1):1–5. 81 2018. doi:10.1038/s41387-018-0046-9. 29795366 PMC5968030

[36] Laursen MF, Dalgaard MD, Bahl MI. Genomic GC-Content affects the accuracy of 16S rRNA gene sequencing based microbial profiling due to PCR bias. Front Microbiol. 2017;8:1934. doi:10.3389/fmicb.2017.01934. 29051756 PMC5633598

[37] Callahan BJ, McMurdie PJ, Rosen MJ, Han AW, Johnson AJA, Holmes SP. DADA2: high-resolution sample inference from Illumina amplicon data. Nat Methods. 2016;13(7):581–583. doi:10.1038/nmeth.3869. 27214047 PMC4927377

[38] Wang Q, Garrity GM, Tiedje JM, Cole JR. Naïve bayesian classifier for rapid assignment of rRNA sequences into the new bacterial taxonomy. Appl Environ Microbiol. 2007;73(16):5261–5267. doi:10.1128/AEM.00062-07. 17586664 PMC1950982

[39] Bolyen E, Rideout JR, Dillon MR, Bokulich NA, Abnet CC, Al-Ghalith GA, Alexander H, Alm EJ, Arumugam M, Asnicar F, et al. Reproducible, interactive, scalable and extensible microbiome data science using QIIME 2. Nat Biotechnol. 2019;37(8):852–857. doi:10.1038/s41587-019-0209-9. 31341288 PMC7015180

[40] Johnsen LG, Skou PB, Khakimov B, Bro R. Gas chromatography - mass spectrometry data processing made easy. J Chromatogr A. 2017;1503:57–64. doi:10.1016/j.chroma.2017.04.052. 28499599

